# The Capricious Character of Nature

**DOI:** 10.3390/life2010165

**Published:** 2012-01-11

**Authors:** Jaana Keto, Arto Annila

**Affiliations:** 1 Department of Biosciences, University of Helsinki, Fi-00014 Helsinki , Finland; E-Mail: jaana.keto@helsinki.fi; 2 Department of Physics, University of Helsinki, Fi-00014 Helsinki, Finland; 3 Institute of Biotechnology, University of Helsinki, Fi-00014 Helsinki, Finland

**Keywords:** evolution, free energy, natural selection

## Abstract

The on-going whole genome sequencing and whole cell assays of metabolites and proteins imply that complex systems could ultimately be mastered by perfecting knowledge into great detail. However, courses of nature are inherently intractable because flows of energy and their driving forces depend on each other. Thus no data will suffice to predict precisely the outcomes of e.g., engineering experiments. All path-dependent processes, most notably evolution in its entirety, display this capricious character of nature.

## 1. Introduction

Did you know that during 2008 in Britain 78.7 per cent of 11- to 15-year-olds visited a library and that 64 self-propelled railroad coaches were imported to the UK? These and numerous other statistical details are provided by the UK National Statistics Office [1], but why are these seemingly random pieces of information about social statuses and economic assets collected? The Statistics Authority, directly accountable to the Parliament, purports to serve the public good. To claim such a fine, yet elusive, objective is unusual today, but undoubtedly it takes a lot of knowledge about the state of affairs to improve a whole nation’s quality of life.

Despite the wealth of information at hand, sometimes decisions will nevertheless result in unexpected outcomes, often countering good intentions. Even minor unknown variables matter greatly when predicting consequences of actions in a complicated system where everything is related to everything else.

The capricious character of socio-economic systems is also a typical trait of biological systems. To tailor an organism to behave in an intended manner, even when based on a whole-cell account of its metabolites and expressed proteins, will every so often end up with surprising results. It seems as if no amount of knowledge would suffice to precisely predict the effects of engineering. Here a question about the fundamentals of unpredictability arises. Could it be that there is some profound principle, inherent to all systems, which ultimately precludes us from attaining certainty in predictions?

## 2. The Natural Principle

There are reasons to regard the similarities between socio-economic and biological systems as not merely analogous, but arising from their identities. Both nations and animates grow and decline along sigmoid curves as well as display concurrent changes in their skewed distributions of assets and populations of diverse species [2]. Unraveling this scale-spanning conundrum calls for general concepts, of which energy is the most essential. Namely, energy can be assigned to everything that exists and thereby place everything in relation to everything else. Then, it is possible to formally describe nature at all levels of its hierarchical organization at a quantum precession as a system that evolves by diminishing energy differences, that is, by consuming the most free energy in the least time.

The natural principle of least-time free energy consumption obtained its mathematical form by the middle of the 18th century, when the naturalistic school devised means to respect the conservation of energy in transformations [3,4] and to express the quest of natural systems for optimal conducts [5], and a century later formal values of energy could be assigned to everything that exists [6]. Nonetheless physics trailed from these general grounds on deterministic and reductionist tracks. Apparently, it is natural to seek security by perfecting predictions rather than understanding the nature of the unpredictable.

## 3. The Cause of Intractability

As a child it was fun to scrape canals in dirt to direct flows of water from one puddle to another. The joy diminished though when another toddler began to drain a common reservoir by carving a bigger duct, thereby leaving my canal with less water. For the worse, the growing current itself carved the competing duct deeper leaving my duct with even less flow. Thus began a race to carve ever deeper canals as the flows themselves naturally kept selecting paths for ever faster drainage. Clearly, the flows and level differences in the network of waterways depended on each other—curiously though—in an intractable way since the probable process itself kept shaping the landscape of brooks.

It turns out from a mathematical analysis that the future course of an elementary waterway system, just as any other energy transduction system, e.g., a system of metabolic pathways, irrespective of the degree of complexity, cannot be predicted in a deterministic manner when there are alternative paths for the energy dispersal [7]. This dilemma appears already in the three-body problem [8] as well as in other problems [9,10] such as protein folding [11]. The non-computable character of nature does not stem from complexity and heterogeneity of the system as such, but since the quantized flows of energy and the level differences of energy as their driving forces depend on each other, there is no way to separate the variables and to solve the dissipative, hence irreversible, equation of motion to know how the system will evolve [12,13,14,15]. In other words, when a system evolves, the natural process itself will change the surrounding boundary conditions due to net quantized flux of energy between the system and its surroundings. Conceivable pathways do not exist *a priori* but the one will form when taken. This path-dependence of a natural process means that the paths cannot be integrated beforehand to make predictions because their boundaries will change when processed. Even a sporadic event is enough to affect the outcome. For example, when a plant happens to catch a photon from insolation, the same photon cannot be absorbed by another plant. Consequent courses of growth for the two plants are affected which, in turn, will influence their future abilities to absorb more photons. Since the quantized flows of energy are not infinitely divisible among alternative paths to consume free energy, equations of motion given in terms of continuous variables are not faithful representations of nature [16]. To this end the concept of action and the principle of least action are appropriate to describe nature and its evolution in quantized terms [5,17].

The naturalistic tenet recognizes no demarcation between animate and inanimate that both display the same scale-independent patterns. Hence, no distinct moment and place or scenario for the emergence of life can be identified either. Moreover, it follows from the inherent intractability of natural processes that no particular path of evolution can be unambiguously traced back from the present to the primordial times [18,19]. Life has its evolutionary history, but due to dissipation also information that would be required to deduce the past unambiguously is invariably lost.

**Figure 1:**
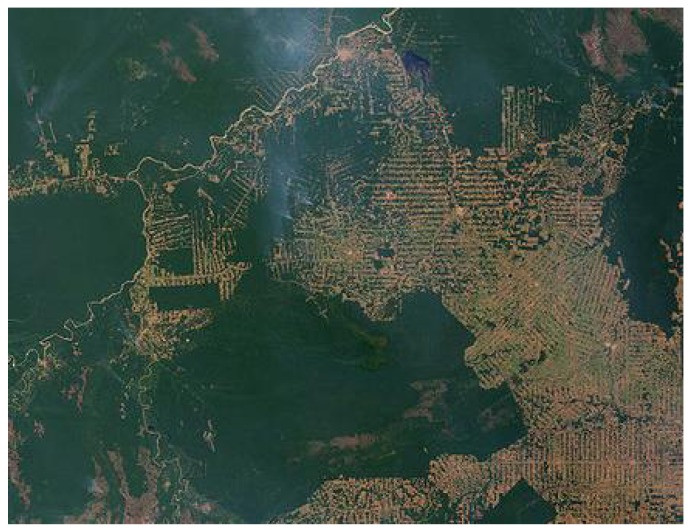
The notion of energy landscape in evolution is exemplified by an aerial view of Amazonas. The age-old stationary forest ecosystem is perturbed where twists of smoke rise from progressive forest fires. Deforestation exemplifies an intractable thermodynamic process where local means of absorbing insolation are demolished, which in turn will affect the surrounding global system. The ensuing global changes will, in turn, impose further changes in the local system, and so on [28].

In order to predict precisely the course of a natural process, it is not enough to describe everything about the system in terms of energetics, because evolution itself is molding the energy landscape which can be pictured as living landscapes of various kinds [20,21,22,23]. These processes are familiar from environmental changes imposed by human activities (Figure 1), but include also changes in socio-economic systems. For example, even revealing information or disinformation about stock exchange will irreversibly intermingle with the energy transduction of the system and contribute to the ambiguity of its future course [24]. Information is physical too [25,26]. Many politicians have learned this characteristic of nature the hard way. Trajectories can be calculated only for systems whose energy remains constant in steady surroundings, and effects can be mapped unambiguously to their causes solely in those systems that have no alternative ways for energy dispersal. For example, when there is no influx or efflux of energy, the orbit of a planet is closed and steady. Likewise, the metabolic cycle of a cell or the nutrient cycle of an ecosystem stays at thermodynamic stationary state, when the influx and efflux remain equal. Thus, only when energy is definite, is there no uncertainty about the fate of the system, but in such cases there are actually no unknowns left to be predicted. We may fear unknowns but also new marvels emerge from the same energy influx [27] as the capricious character of nature.

## 4. Conclusions

The above-described physical portrayal of nature does not deny that knowing more of a biological or social system will make a better forecast but asserts that a perfect prediction is an illusion. On the other hand, the naturalistic tenet reveals the public good not as an imaginary esteem of a government, but assigns it with a quantitative measure. To be exact, that measure is of entropy [29].
